# A Sea-Surface-Zoning Method Based on Fractal Characteristics

**DOI:** 10.3390/s22134761

**Published:** 2022-06-24

**Authors:** Huaxing Kuang, Luxi Yang

**Affiliations:** 1 School of Information Science and Engineering, Southeast University, Nanjing 211189, China; lxyang@seu.edu.cn; 2 Nanjing Marine Radar Institute, Nanjing 211153, China

**Keywords:** sea surface zoning, radar theory, fractal characteristics

## Abstract

In this paper, a criterion for sea surface zoning based on the fractal characteristics of disturbances is demonstrated. To improve radar detection performance in heavy-tailed sea clutter, a multitude of sea clutter models and corresponding optimum and suboptimum detectors have been designed in recent years. However, there are cases where these models and detectors become insufficient to describe the coexistence of noise and sea clutter. The commonly used signal-to-clutter ratio (SCR) can hardly serve as an indicator revealing which kind of disturbances dominate in a certain area since it is difficult to decide the level of SCR at which sea clutter or noise exceeds the other. Therefore, it is necessary that a set of rules reflecting essential differences between sea clutter and noise are proposed to tell areas where sea clutter dominates, areas where sea clutter and noise coexist and areas where noise dominates. Analyzing fractal characteristics of disturbances, we consider the Hurst exponent *H* as a feature distinguishing sea clutter and noise from each other. A modified Sigmoid function is employed to model the variation in H with range bins, and the derivative of the function helps to formulate a set of rules for sea surface zoning.

## 1. Introduction

Sea clutter refers to the back-scattered returns from a patch of the sea surface illuminated by a radar pulse. Exploring and characterizing its nature is of great significance for target detection within sea clutter, and since the invention of radar, efforts have been taken to establish appropriate models describing sea clutter and design corresponding target detectors.

For many years, when radar resolution is low, sea clutters have been considered a kind of Gaussian disturbance. However, for modern radar systems operating at low grazing angles and with high-resolution capabilities, sea clutter echoes tend to be heavy-tailed, exhibiting a deviation from normality [1,2,3,4,5]. The deviation from normality leads to spikier sea clutters, and the spikes are often mistaken for targets. In this situation, traditional cell-averaging CFAR (CA-CFAR) and its derivations based on Gaussian sea clutter suffer significant capability loss. Therefore, the emergence of non-Gaussian sea clutter calls for novel statistic models and corresponding optimum and suboptimum detectors.

Numerous theoretical models are proposed to fit the amplitude statistics of non-Gaussian sea clutter, including the log-normal, the Weibull [1,2], etc. In recent years, more and more attention has been paid to a family of compound Gaussian models. The compound Gaussian model claims that a sea clutter random variable is the product of random variables, namely the square root of texture component and speckle component [6]. Texture represents the long-range modulation of the sea surface and describes the underlying mean value of the data, while speckle models the local modulation. Speckle is considered a complex Gaussian process with zero mean and unit variance, while the distribution of texture determines the category of compound Gaussian model the data belong to. If texture satisfies Gamma distribution, the sea clutter is a K-distributed random variable [4]; if texture satisfies inverse Gamma distribution, the sea clutter is a generalized Pareto-distributed random variable [7]; and if texture satisfies inverse Gaussian distribution, the sea clutter is an inverse-Gaussian–compound-Gaussian (IGCG)-distributed random variable [8]. Studies have revealed that the compound Gaussian model is a good fit for heavy-tailed sea clutter data. Corresponding optimum and suboptimum detectors have also been developed for these compound Gaussian models.

In some cases, noise cannot be neglected in target detection, especially when clutter is relatively weak. In this situation, noise must be considered in compound Gaussian models and the predesigned detectors must be modified. This is a challenging field in target detection within sea clutter since it is hard to model the mixed disturbance of sea clutter and noise with an analytic expression. Some tentative efforts have been taken to tackle the detection problem with a mixed detector [9]. However, the mixed detector is after all an approximation to optimum and predesigned suboptimum detectors, which inevitably suffers capability loss. Therefore, a zoning method is required to partition the sea surface into areas where sea clutter dominates, areas where noise dominates and areas where sea and noise coexist.

The criteria for sea surface zoning have always been a major concern in practice. While clutter–noise ratio (CNR) is a direct index reflecting the components of disturbance, it is not a good criterion for sea surface zoning since it is hard to determine whether clutter dominates an area given a value of CNR. Zhou Ming et al. describe a sea-surface-zoning method [10] based on grazing angles, but it is an empirical one and lacks concrete physical meaning. In this paper, we propose a novel sea-surface-zoning method based on fractal characteristics of disturbance. The article is organized as follows: Section 2 provides a description of the fractal characteristics of sea clutter and how we extract certain fractal characteristics from the data. In Section 3, numerical analysis is shown to propose our criteria. Finally, in Section 4, a conclusion is reached.

## 2. Theoretical Analysis

In the 1970s, fractal theory was introduced by Mandelbrot [11] to describe natural rough structures such as coastlines, tree branches, lightning, and so forth. Since sea clutter is a result of a scattering phenomenon involving electromagnetics and the sea surface, it is natural to consider the possibility of applying fractal theory to sea clutter research. T. Lo et al. initially analyze some real sea clutter data and claim they are fractal with the dimension being about 1.75 [12]. Later, according to fluid theory, the sea surface was modeled by a limited-band Weistrass–Mandelbrot function, which is fractal [13]. Mathematical deduction reveals that the real part and imaginary part of the scattered sea clutter echoes retain the fractal characteristics of the sea surface.

In this paper, we demonstrate that a sea clutter data series is fractal based on the random walk model. We suppose $X=\{X_{i},i=1,2,\ldots ,N\}$ represents a real sea clutter data series with mean μ and variance $\sigma^{2}$, and then derive a new increment series $x=\{x_{i},i=1,2,\ldots ,N\}$ by subtracting μ from the original series. The random walk process can be defined as

(1) $$y(n)=\sum_{i=1}^{n}x_{i}$$

If the following relation is satisfied

(2) $$F(m)=<|y(n+m)-y(n){|}^{2}{>}^{1/2}\sim m^{H},$$

then we consider the sea clutter data series as fractal. The notation |˙| represents the 2-norm, <˙> represents mean value, and *H* is the Hurst exponent of a fractal.

Fractal theory is an abstraction of natural phenomena. In practice, Formula (2) holds only in an interval [*m_min_*, *m_max_*] for scale m, which is called a scale-invariant interval. That means it is meaningful to analyze fractal characteristics of a data series only in this interval for scale m. By plotting the log_2_*F*(*m*)~log_2_*m* curve, it is not difficult to determine the scale invariant interval [*m_min_*, *m_max_*] and the Hurst exponent *H*.

When the Hurst exponent *H* = 1/2, the increment process *x* is a Brownian motion. Generally, the Hurst exponent of a natural process is not 1/2, and the corresponding increment process *x* is a fractional Brownian motion.

## 3. Numerical Demonstration

**Figure 1:**
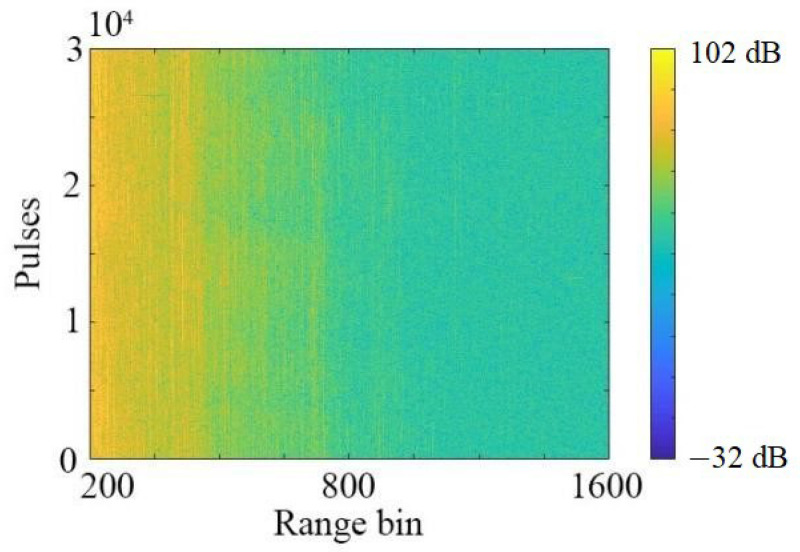
Real sea clutter data.

The real sea clutter data for analysis are shown in Figure 1, with 30,000 pulses and 1601 range bins. The radar is placed at a seashore with an altitude of 20 m and the beamwidth is 0.6 degrees. The data are collected under the steering mode.

**Figure 2:**
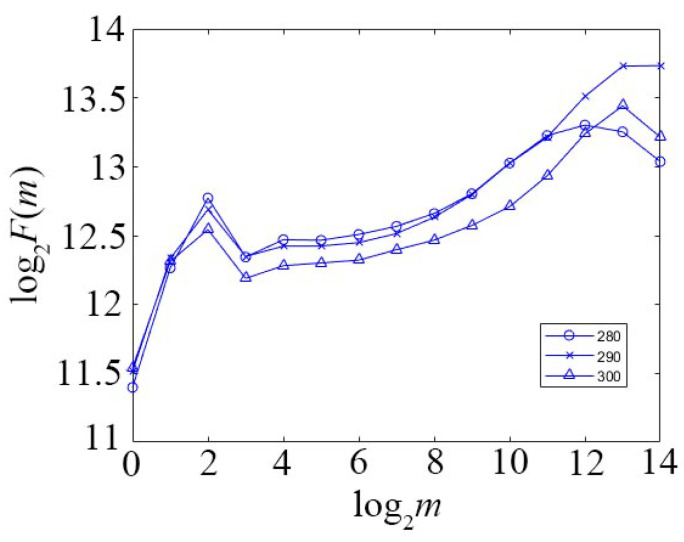
log_2_*F*(*m*)~log_2_*m* curve for various range bin.

In order to evaluate the scale-invariant interval, the method based on the random walk model in Section 2 is employed to plot the log_2_*F*(*m*)~log_2_*m* curve. As shown in Figure 2, in a scale interval [2^3^, 2^12^], log_2_*F*(*m*) exhibits good linearity. By linear fitting, the slope, i.e., the Hurst exponent *H*, can be evaluated.

**Figure 3:**
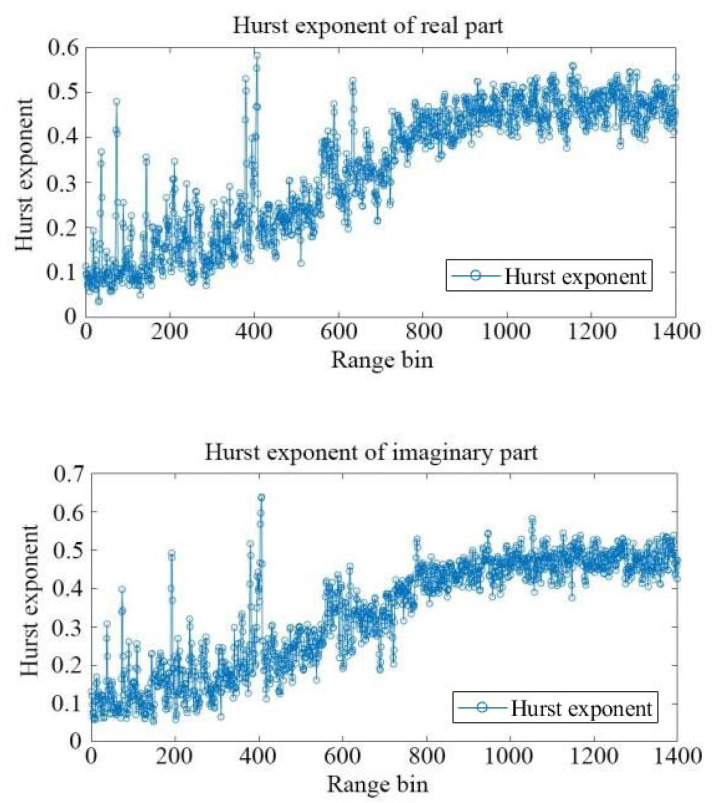
The Hurst exponent *H* of real part and imaginary part of sea clutter for each range bin.

According to Jian Guan et al. [14], the Hurst exponent of sea clutter data in the real part and imaginary part is identical for certain range bins. Subsequently, the Hurst exponent *H* of sea clutter in the real part and imaginary part for each range bin is evaluated by the least square method. The results are shown in Figure 3.

As shown in Figure 3, the general trend and spike positions for the real and imaginary parts are almost identical. The slight difference is due to estimation error of the algorithm. The spikes of the curves correspond to targets hidden in the sea clutter. This is also the basis for radar target detection based on fractal characteristics [14]. The general trend intuitively undergoes three stages. In the first stage, the Hurst exponent is relatively stable. The second stage, starting from about the 200th range bin, witnesses an increase in the Hurst exponent, and the acceleration seems to increase at first and then diminish. In the third stage, starting from about the 1000th range bin, the Hurst exponent curves become stable again. The change in the curve with range bins is due to the increased power ratio of noise. It is evident that in the third stage, the Hurst exponent is 0.5. That means the data in these range bins are Brownian noise. It also makes sense that the stability in the first stage reflects the dominance of sea clutter. Similarly, the increase in the second stage means data in these range bins are a mixture of sea clutter and noise.

The curve of Hurst exponent versus range bin is an S-shaped one. A useful mathematical function called the Sigmoid function is often used to model the S-shaped curve. The Sigmoid function is

(3) $$f(x)=\frac{1}{1+e^{-x}},$$

Specifically in order to describe the trend shown in Figure 3, we design a modified Sigmoid function as follows:

(4) $$f(x)=\frac{c}{1+e^{-a(x+b)}}+d,$$

**Figure 4:**
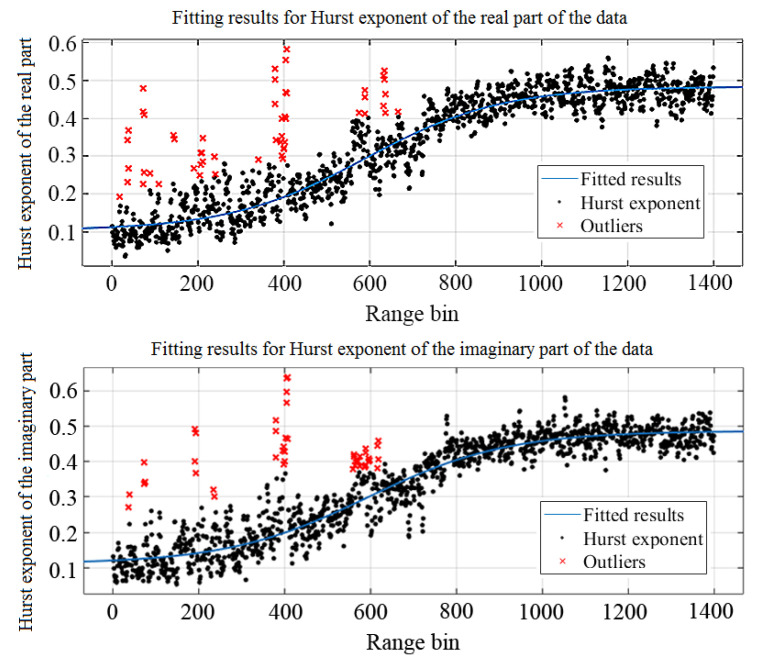
Fitting results with modified Sigmoid function.

The Levenberg–Marquardt algorithm [15,16] is employed to fit the data shown in Figure 3 using a curve-fitting tool. According to the fitting results, the parameters of the modified Sigmoid function are listed as follows (see Table 1):

It is evident from the table that for the real part and imaginary part of the data, the fitted modified Sigmoid functions are almost the same, which is in accordance with [14] as previously mentioned. The fitting results are displayed in Figure 4. The RMSEs of fitting for the Hurst exponent of the real and imaginary parts are both about 0.04, which is close to 0. The R-squares of fitting for the Hurst exponent of the real and imaginary parts are both about 0.92, which is close to 1. This means the modified Sigmoid function is a good approximation of the curve trend with range bins. It is worth noting that, in practice, there might be other mathematical models fitting the shaped curve well, even with better RMSE and R-squares. The Sigmoid function is just the most famous and simple S-shaped model which serves as an example here.

In subsequent analysis, the parameters acquired according to the real part of the data are employed.

The derivative of the modified Sigmoid function is

(5) $$f^{\prime }(x)=\frac{ace^{-a(x+b)}}{{\left(1+e^{-a(x+b)}\right)}^{2}},$$

**Figure 5:**
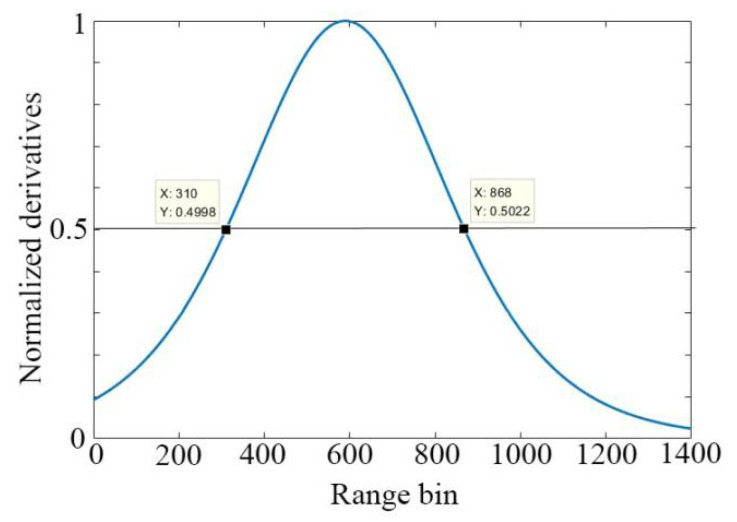
The derivative of the modified Sigmoid function.

**Figure 6:**
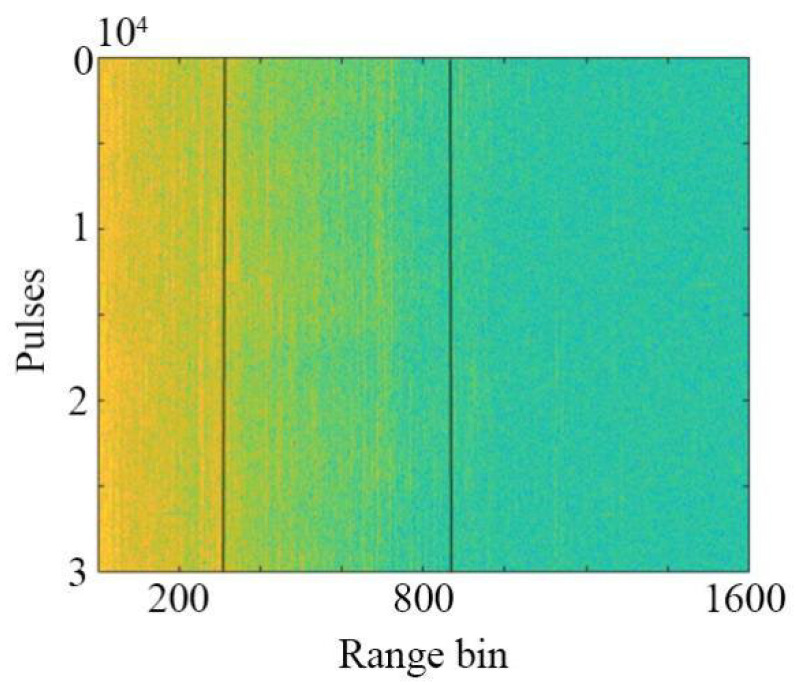
Sea-surface-zoning results with T = 0.5.

The plotting of the derivative is shown in Figure 5. A threshold T can be set as the criteria for sea surface zoning. Analogous to the definition of half peak width in physics, in this context, we empirically set the threshold T = 0.5, the area where clutter dominates is range bin 1–310, the area where clutter and noise coexist is range bin 311–868, and the area where noise dominates is that beyond range bin 869. The sea surface zoning is demonstrated in Figure 6.

According to the sea-surface-zoning result, a set of detection rules can be applied for different areas. An example is given as below:

1. For the area where clutter dominates, any detector design in the background of sea clutter is applicable.
2. For the area where noise dominates, traditional CA-CFAR and its derivations are satisfying enough.
3. For the area where clutter and noise coexist, a mixed detector as proposed in [9] is an option.

## 4. Conclusions

Sea surface zoning helps to improve the performance of coherent detectors. However, for a long time, the criteria for sea surface zoning have been unclear. As quantities indicating various natural and artificial processes, fractal characteristics are employed in this article to distinguish clutter, clutter plus noise and noise. The criterion based on fractal characteristics is reliable because it can reflect some essential nature underneath the clutter echoes and noise. The sea-surface-zoning method proposed in this article promises to tailor target detection methods within sea clutter. Further research may focus on the detector design in areas where sea clutter and noise coexist.

## Figures and Tables

**Table 1:** Parameters of the fitted modified Sigmoid functions.

Parameters	For Real Part	For Imaginary Part
a	−0.0063	−0.0061
b	−589.50	−593.50
c	−0.38	−0.38
d	0.48	0.49

## Data Availability

Not applicable.
